# Bibliometric analysis of publications on research into cotton leaf curl disease

**DOI:** 10.15190/d.2020.6

**Published:** 2020-06-07

**Authors:** Ayyaz Khan, Darya Khan, Fazal Akbar

**Affiliations:** ^1^Center for Biotechnology and Microbiology, University of Swat, KP, Pakistan

**Keywords:** Bibliometric, cotton leaf curl disease, publications, research.

## Abstract

Cotton leaf curl disease (CLCuD), caused by viruses of the family Geminiviridae (genus Begomovirus), is of great concern for cotton production worldwide. The aim of the study was to characterize and quantify the worldwide scientific output of CLCuD research using bibliometric analysis. PubMed, Google Scholar and Scopus search engines were used to extract available data from 1901 to July 2017. A total of 854 CLCuD-related published documents were identified. Most of the documents were published in the form of original research articles (644, 75.4 %) and English was the main language of publication (807, 94 %). The results demonstrate that the study of CLCuD exhibits an overall increasing trend from 1991 to 2017, with the highest number of articles published in 2013. The top 10 countries in terms of absolute research output (number of publications) on this subject were Pakistan (217; 25.40%), India (161; 18.85%), the United States of America (USA; 122; 14.85%), China (85; 9.95%), United Kingdom (57; 6.67%), Sudan (31; 3.62%), Israel (14; 1.63%), Spain (13; 1.52%), Australia (11; 1.28%), Saudi Arabia (9; 1.05%) and Iran (9; 1.05%). Pakistan’s most important collaborator was United States of America, followed by China. Noteworthy, not one of the papers listed here was the result of scientific collaboration between India and Pakistan. The total number of citations for all the publications was 3174, with an average of 3.71 citations per publication. The h-index for all extracted data related to CLCuD was 91. The top h-index was achieved by Pakistan (54) followed by the United Kingdom (43), the USA (41) and India (39). The National Institute for Biotechnology and Genetic Engineering (NIBGE), Faisalabad, ranked the first in the top 10 list of the most productive institutes. This bibliometric analysis highlights the leading role of Pakistan, India and the USA in research on CLCuD and points out that the initiation of a collaboration between Pakistan and India may have a significant impact on the research output and progress.

## 1. Introduction

Cotton leaf curl disease (CLCuD) is a severe disorder of cotton (particularly *Gosypium hirsutum *but also other* Gossypium *species) transmitted by the whitefly *Bemisia tabaci^*^1^*^.* The disease was first reported in Nigeria in 1912, affecting *G. vitifolia *and *G. Pervianum^2^* and then in Tanzania in 1926 and Sudan in 1934^3^. In Asia, CLCuD was reported in Pakistan in 1967 in the vicinity of Multan^4^. Initially, the disease in Pakistan was sporadic and caused only minor economic losses. However, in 1987, the disease became epidemic in Pakistan and, by the early 1990s, it spread into northwestern India and Southern China^1,5^. CLCuD-affected cotton plants have characteristic symptoms that include downward or upward leaf curling, vein thickening, darkening of veins, enations and, dependent upon cotton variety, the formation of leaf-like enations on the veins on the undersides of the leaves^1,5^. CLCuD has a severe effect on the growth of cotton plants. Particularly reduces the yield and quality of lint (cotton fiber)^6^.

The causal agent of CLCuD was for the first time identified as a virus in 2001^7^. More recently, the disease has been shown to be caused by begomoviruses (*B. tabaci* transmitted viruses of the genus *Begomovirus*, family *Geminiviridae*) in association with specific satellites known as betasatellites, which are true satellite DNAs entirely dependent on their helper viruses for replication and transmission. The disease complexes in Africa and Asia are distinct. The disease in Africa has not been extensively investigated and CLCuD affected cotton has been shown to be caused by the begomovirus *Cotton leaf curl Gezira virus* and the betasatellite *Cotton leaf curl Gezira betasatellite^8^.* A number of distinct begomoviruses have been shown to cause CLCuD in Asia, in association with the betasatellite Cotton leaf curl Multan betasatellite, the most important of which are *Cotton leaf curl Multan virus* and *Cotton leaf curl Kokhran virus^7,9^.* A number of other viruses have been sporadically identified in CLCuD affected cotton in Asia. However, these are thought not to be significant^10,11^. For both the African and Asian CLCuD complexes the disease symptoms are associated with the betasatellites rather than the virus(es)^8,12^.

Bibliometrics is the statistical analysis of published documents. This is used for the qualitative and quantitative analysis of published literature. Bibliometric analysis is used in many different research areas to explore the productivity and impact of a specific research field or specific researchers, and to evaluate the research activities in many scientific fields. Over the last decade, bibliometric analyses have been conducted for many human infectious diseases^13-17^. However, no such studies have been conducted on the scientific research output for CLCuD. Thus, the aim of this study is to characterize and quantify the research productivity on CLCuD at the global level using bibliometrics. Our study provides an overview of the previous research conducted on CLCuD and identifies the leading countries and institutes involved in CLCuD research. The results identify possible future collaborations which may have a significant impact on increasing the research output and progress on the subject.

## 2. Methods

### 2.1 Study design

This study was based on bibliometric analysis^13-17^. Major databases, such as PubMed, Google Scholar and Scopus were employed as a source for our research on CLCuD/CLCuV. Collected data (publications) was analysed using various statistical tools.

### 2.2 Search strategy 

The standardized search approach was used for bibliometric analysis^13^. This was based on the use of the keywords “Cotton leaf curl disease” and “Cotton leaf curl virus” in the title, abstract, and keyword field, to obtain CLCuD/CLCuV-related research, as used by Sa’ed for bibliometric analysis of dengue research^13^. Data related to CLCuD/CLCuV was collected from 1901 to July 31, 2017. The cumulative data was used to obtain: (a) total number of documents, (b) international collaboration, (c) authorship pattern, (d) citation received, (e) journal name in which research is published, (f) country name of authors and institutions, (g) year of publication, (h) impact factor, (i) *h*-index and (j) language of publication.

### 2.3 Data analysis

The data downloaded from the databases were organized in Microsoft Excel 2016 and then used for further analysis^13^. The top 10 ranked countries were determined by the standard competition ranking procedure (1–2–2–4 rule). This competition ranking assigns the same rank to the values that are identical and then a gap is left within the ranking list. For the percentage, sum, average and frequency determination descriptive statistics were used. The impact factor (IF) and* h*-index were used to assess the quantity and quality of the research output. The *h*-index in bibliometric analysis shows the scientific output of a country, researcher and organization etc. The *h*-index covers both the number of publications (quantity) and number of citation (impact). The impact factors for journals were obtained from the Journal Citation Reports (JCR), ranking: 2016/ 2017. The analysed data were then presented in tables and graphical form.

## **3. ** Results

A total of 854 documents related to CLCuD/CLCuV, published between 1901 and July 2017, were identified. These documents consisted of 644 (75.4 %) original research articles, 94 (11 %) review articles, 85 (9.9 %) books, proceeding papers and notes, and 31 (3.7 %) editorial letters (Table 1).

**Table 1:** Type of articles published on CLCuD (n =854)

Rank	Type of article	Number of articles (percentage of articles)
1	Original research articles	644 (75.4%)
2	Review articles	94 (11%)
3	Books, proceeding papers, and notes	85 (9.9%)
4	Editorial letters	31 (3.7%)

The documents were published in more than 22 languages, the most common being English (807, 94 %), Chinese (18, 2 %), Spanish (16, 1.8 %) and French (11, 1.2 %) (Table 2).

**Table 2:** Language of published research articles on CLCuD (n =854)

Rank	Language	Number of articles (percentage of articles)
1	English	807 (94 %)
2	Chinese	18 (2 %)
3	Spanish	16 (1.8 %)
4	French	11 (1.2 %)

**Figure 1:**
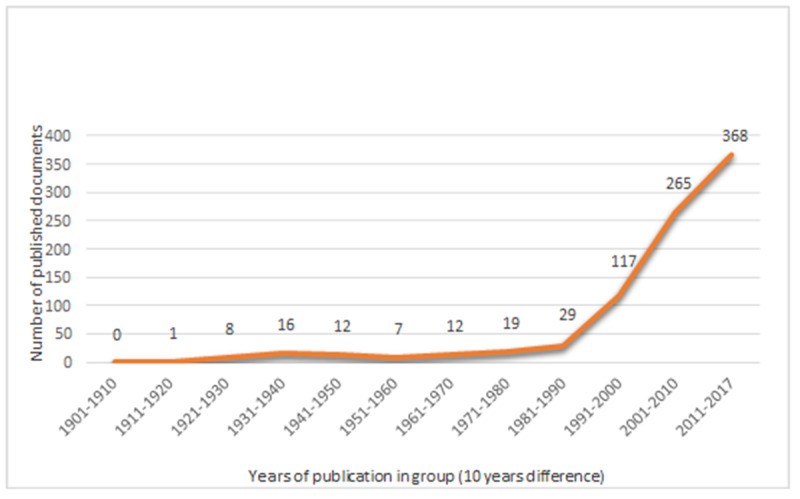
Number of research publication on cotton leaf curl disease/cotton leaf curl virus between 1901 and July 2017 The data shown for 2017 is incomplete, since the data were collected only up to July 2017. The graph presents the publications years (grouped by decade, x-axis) versus the number of publications (y-axis).

Prior to 1912, there were no publications on CLCuD. The first research article on CLCuD was published in Nigeria in 1912. From 1912 to 1950 the numbers of publications on CLCuD/CLCuV were low and slowly increased until 1990, when the number of publications increased dramatically (Figure 1). This coincides with the outbreak and subsequent epidemic spread of CLCuD in Pakistan. Most of the CLCuD/CLCuV related documents were published from 2001 to 2017, with the highest number of publications in the year 2013. The total documents published from 2001 to July 2017 were 654 (76.6%). The data shown for 2017 is incomplete, since the data was collected only up to July 2017. The publications grouped in decades are shown in Figure 1.

The 10 most prolific publishing data on CLCuD/CLCuV, their *h*-index, and their collaboration with other countries are shown in Table 3. The published documents related to CLCuD were identified from more than 55 countries. The leading countries in terms of absolute research production (number of publications) were Pakistan (217; 25.40%), India (161; 18.85%) and the United States of America (USA; 122; 14.85%). The USA, a county in which CLCuD does not occur, leads in terms of published documents in collaboration with other nations (5.73%), followed by Pakistan (4.91%) and India (3.1%). Scientists from the USA had collaborations with 19 multinational researchers, followed by Pakistan and India who had research collaboration with 16 and 13 countries, respectively. Iran had only two research collaborations with the other nations (Table 3). This bibliometric analysis demonstrates that, not surprisingly, Pakistan and India have played a significant role in CLCuD research (Table 3). The total number of citations for all publications was 31,749. The average of these citations was 3.71 per publication. The *h*-index for all the extracted data related to CLCuD/CLCuV was 91. The top *h*-index was achieved by Pakistan (54) followed by the United Kingdom (43), the USA (41) and India (39) (Table 3).

**Table 3:** Published research on CLCuD from 1901 to July 2017, country ranking (n =854) SCR – standard competition ranking; ^*^ are the countries with the same number of publications.

SCR	Countries	No. of articles (%)	h-index	No. of countries collaborated with	No. of articles (percentage of publications) in collaboration with other nations
1	Pakistan	217(25.40%)	54	16	42(4.91%)
2	India	161(18.85%)	39	13	27(3.1%)
3	USA	122(14.28%)	41	19	49(5.73%)
4	China	85(9.95%)	10	8	17(1.99%)
5	United Kingdom	57(6.67%)	43	9	19(2.22%)
6	Sudan	31(3.62%)	11	10	7(0.81%)
7	Israel	14(1.63%)	8	3	4(0.46%)
8	Spain	13(1.52%)	6	6	4(0.46%)
9	Australia	11(1.28%)	6	6	5(0.58%)
10*	Saudi Arabia	9(1.05%)	3	5	6(0.70%)
10*	Iran	9(1.05%)	7	2	3(0.35%)

**Figure 2:**
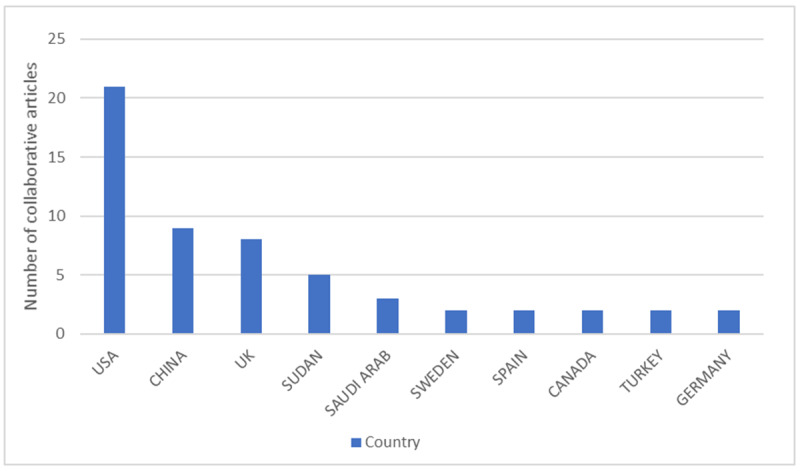
The top 10 countries collaborating with Pakistan on the CLCuD Pakistan tops the list in terms of total number of publications on the studied subject. Most of these documents were published in collaboration with the other nations.

Researchers in Pakistan topped the list in terms of total number of publications (217). However, most of these documents were published in collaboration with researchers from second countries. Pakistan had the maximum number of publications in collaboration with the USA (21), China (9), United Kingdom (8), Sudan (5), and the Kingdom of Saudi Arabia (3) (Figure 2).

The most frequently cited articles on CLCuD/CLCuV are listed in Table 4. The most highly cited article, detailing the identification of the complex causing the disease^7^ in the journal ‘’*Virology*’’ (IF=3.35), had 400 citations. R.W. Briddon, and S. Mansoor were the most prolific authors publishing research on CLCuD/CLCuV authoring the majority of the articles in the top 10 list of the most frequently cited articles.

**Table 4:** Top 10 most cited research articles related to CLCuD Citations are Google Scholar-based; SCR – standard competition ranking.

SCR	Title	Year of Publication	Journal	Citations in July 2017	**Citations in May 2020**	**Reference**
1	Identification of DNA components required for induction of cotton leaf curl disease.	2001	Virology	400	478	^7^
2	Identification of a novel circular single-stranded DNA associated with cotton leaf curl disease in Pakistan.	1999	Virology	198	373	^18^
3	Four DNA-A variants among Pakistani isolates of cotton leaf curl virus and their afﬁnities to DNA-A of geminivirus isolates from okra.	1998	Journal of General Virology	196	217	^19^
4	Cotton leaf curl virus disease.	2000	Virus Research	193	261	^20^
5	Cotton leaf curl disease is associated with multiple monopartite begomoviruses supported by single DNA β.	2003	Archives of Virology	150	332	^9^
6	Cotton leaf curl disease, a multicomponent begomovirus complex.	2003	Molecular Plant Pathology	116	150	^21^
7	Detection and relationships of cotton leaf curl virus and allied whitefly-transmitted geminiviruses occurring in Pakistan.	1997	Annals of Applied Biology	110	127	^5^
8	Genetic variability of natural populations of cotton leaf curl geminivirus, a single-stranded DNA virus.	1999	Journal of Molecular Biology	104	119	^22^
9	Transgenic tobacco expressing geminiviral RNAs are resistant to the serious viral pathogen causing cotton leaf curl disease.	2003	Archives of Virology	93	240	^23^
10	Clones of cotton leaf curl geminivirus induce symptoms atypical of cotton leaf curl disease.	2000	Virus Genes	92	107	^24^

The most productive institute, in terms of research publications, was the National Institute for Biotechnology and Genetic Engineering (NIBGE, Faisalabad, Pakistan) with 62 (7.25%) of the published documents on CLCuD research (Table 5). This was followed by Punjab Agriculture University (Ludhiana, India) and the University of Arizona (Arizona, USA) with 33 (3.86%) and 31 (3.62%) published articles on CLCuD, respectively. The University of Queensland (Australia) and King Abdul-Aziz University (Saudi Arabia) were ranked at the same position (number 10) in the top 10 list.

The 10 most prolific journals for publishing CLCuD-related material are presented in Table 6. Documents related to CLCuD were published in more than 80 journals, with the *Journal of Cotton Research and Development *publishing the most articles (196, 22.95%).

**Table 5:** Top 10 most productive institutions publishing research on the CLCuD SCR – standard competition ranking.

SCR	Institute, Country	No. of documents (percentage of documents)
1st	National Institute for Biotechnology and Genetic Engineering Faisalabad, Pakistan	62(7.25%)
2nd	Punjab Agriculture University, Ludhiana, India	33 (3.86%)
3rd	University of Arizona, Tucson, USA	31 (3.62%)
4th	Zhejiang University, Hangzhou, China	26 (3.04%)
5th	Scottish Crop Research Institute@, Invergowrie Dundee, UK	22 (2.57%)
6th	Agriculture Research Corporation, Wad Medani, Sudan	13 (1.52%)
7th	Department of Entomology, Volcani Center, Bet Dagan, Israel	7 (0.81%)
8th	University of Malaga, Spain	6 (0.70%)
9th	Shiraz University, Iran	3 (0.35%)
10th	The Queensland University, Australia.	2 (0.23%)
10th	King Abdul-Aziz University, Saudi Arabia	2 (0.23%)

**Table 6:** The 10 journals publishing research on the CLCuD SCR – standard competition ranking.

SCR	Journal	IF	No. of publications (percentage of publications)
1st	Journal of Cotton Research and Development	NA	196 (22.95%)
2nd	Journal of Cotton Sciences	0.65	98 (11.47%)
3rd	Virus Research	2.628	62 (7.25%)
4th	Archives of Virology	2.058	60 (7.02%)
5th	Virus Genes	1.431	47 (5.50%)
6th	Virology	3.353	42 (4.91%)
7th	Nature	40.137	28 (3.27%)
8th	Archives of Plant Pathology and Plant Protection	0.39	24 (2.81%)
9th	Journal of General Virology	2.838	19 (2.22%)
10th	Asian Journal of Plant Sciences	NA	17 (1.99%)

## 4**. ** Discussion

CLCuD is caused by a group of geminiviruses (genus *Begomovirus*) and is the major limiting factor for the production of cotton on the Indian sub-continent. The study described here employed a bibliometric approach to analyze the research output on CLCuD. Bibliometric analysis such as this has previously been done for several human infectious diseases^13^. This is the first study of this nature to employ bibliometrics to investigate CLCuD research output.

The study showed a steadily increasing trend in the number of publications on CLCuD research in the last few decades. The findings also show that Pakistan, India and USA play the leading role in the area of CLCuD research. The high research output in Pakistan and India, the two countries most severely affected by the disease, is almost certainly due to the effects the disease has on their economies and the high output reflects spending on research to find an answer to the problem. The prominent place of researchers from the USA, likely reflecting funding for research on the topic, may be due to the fear that CLCuD could spread to the cotton growing areas of the USA. Most of the published documents were in the form of original research articles and English was the most common language used.

Scientists from Pakistan also published most articles in partnership with collaborators from the USA. Although Pakistan and India were the two main contributors to CLCuD research, being the two countries most seriously affected by CLCuD, not one article identified here was a result of collaboration between scientists in these two countries. This suggests that partnerships between scientists of these two countries could significantly improve CLCuD research.

NIBGE is the leading institute in CLCuD research, with the highest research output. This is due to the contributions of two leading scientists in the field, R.W. Briddon and S. Mansoor, at this institute. The “*Journal of Cotton Research and Development*” was the leading journal with respect to the number of published articles on CLCuD. However, this journal does not have an IF, which may explain why articles from this journal do not appear in the list of most highly cited articles.

The study presented here is the first attempt to evaluate both the quality and quantity of CLCuD research efforts. However, the current study still has certain constraints. The major limitation is that only few databases were used to extract the literature related to CLCuD. The other limitation is that use of the terms “CLCuD” and “CLCuV” may not identify all related documents. So, papers such as the one from Briddon *et al*.^25^which do not contain the search terms in either the title or abstract, but nevertheless made significant contributions to research in the field, would not be identified. Thus, there is a need to further refine the search terms to identify relevant material.

## 5**. ** Conclusions

Based on 854 CLCuD published documents extracted from the databases, this investigation provided a comprehensive review of the literature in the field of CLCuD research. The analysis demonstrated that the quantity and quality of literature/research related to CLCuD has significantly increased during the last decades. Pakistan, India, and USA were the most productive countries in the field. USA and Pakistani scientist preferred to develop collaboration with the multinationals. Further multinational collaborations, such as the initiation of collaborations between Pakistan and India, may have a significant impact on the research output and progress. in CLCuD research. Scientists in the countries where CLCuD is epidemic/endemic need to take a leading role and promote applied research projects in the field of CLCuD, in order to control this important agriculture-related problem.

## KEY POINTS

◊ Our study demonstrated a steadily increasing trend in the number of publications on CLCuD research in the past few decades.

◊ This bibliometric analysis highlights the leading role of Pakistan, India and the USA in the research on cotton leaf curl disease

◊ A partnership between scientists of India and Pakistan could significantly impact CLCuD research’s output. Although Pakistan and India (most seriously affected countries by CLCuD) were the two main contributors to CLCuD research, not one article identified here was a result of collaboration between their scientists.
